# Supplementary material for: DMRforPairs: identifying Differentially Methylated Regions between unique samples using array based methylation profiles
Source: BMC Bioinformatics. 2014 May 15;15:141. doi: 10.1186/1471-2105-15-141 (PMC4046028; doi:10.1186/1471-2105-15-141)
Supplement: Additional file 2 — DMRforPairs output for the comparison of A431-MCF7 and NA17018-NA17105. Please start from the HTML files in each folder. Available via the BMC Bioinformatics website. [file 1471-2105-15-141-S2.zip › 1394847754114233_MOESM2_ESM/A431_MCF7/figures/33425.pdf]

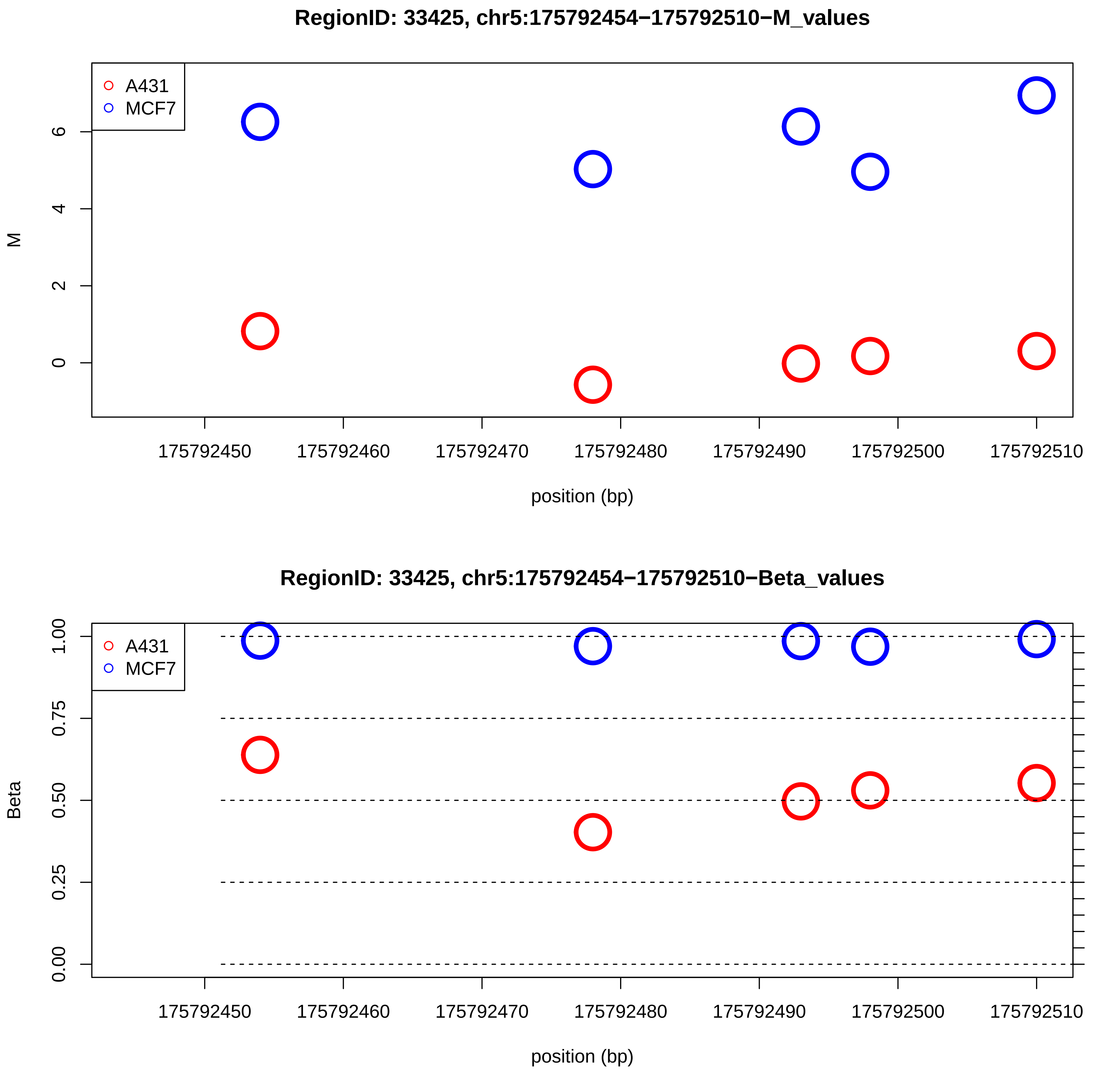

RegionID: 33425, chr5:175792454−175792510−M_values
A431
MCF7
M
0
2
4
6
175792450
175792460
175792470
175792480
175792490
175792500
175792510
position (bp)
RegionID: 33425, chr5:175792454−175792510−Beta_values
A431
MCF7
Beta
0.00
0.25
0.50
0.75
1.00
175792450
175792460
175792470
175792480
175792490
175792500
175792510
position (bp)

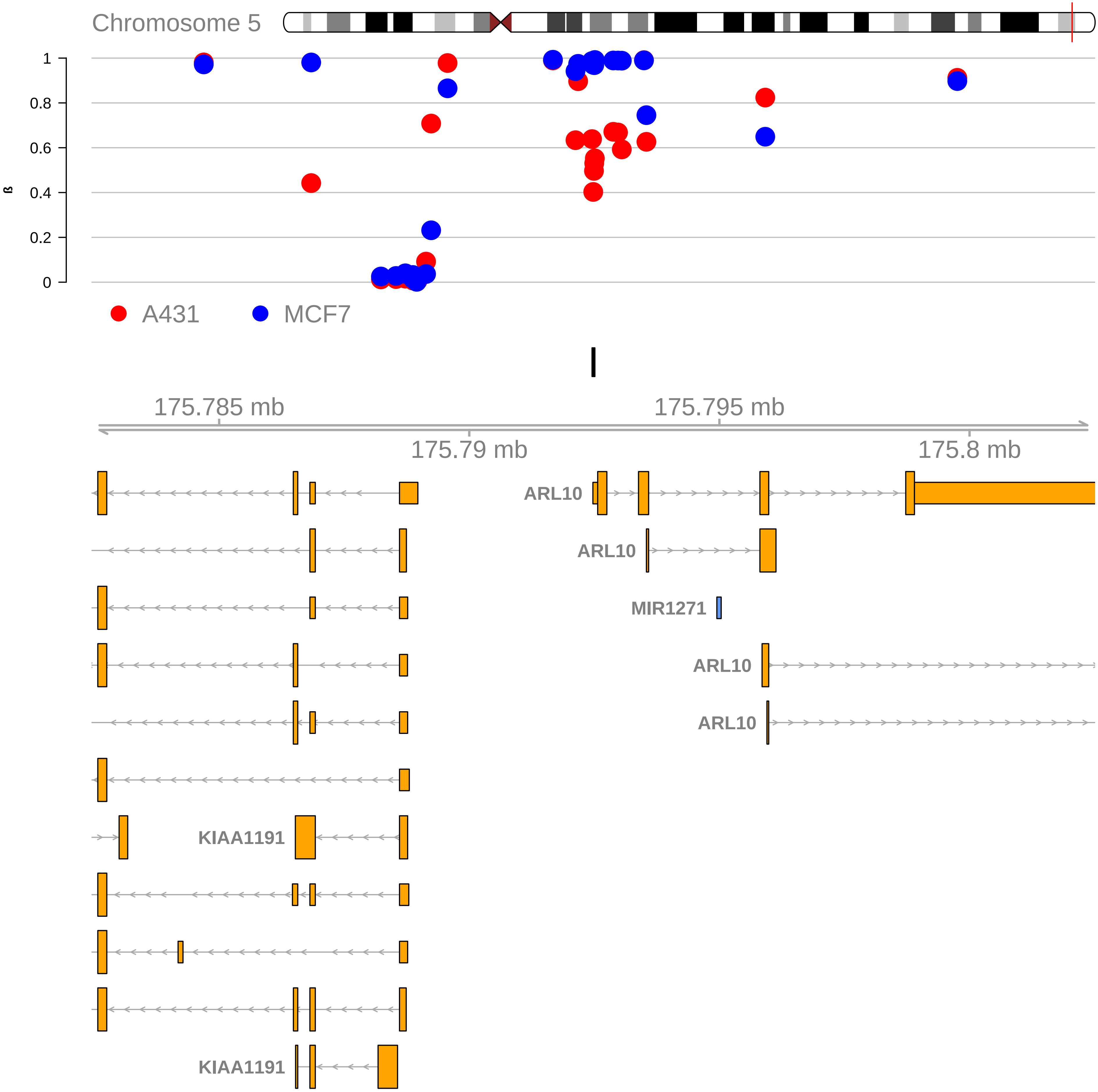

Chromosome 5
1
0.8
0.6
0.4
0.2
0
ß
A431
MCF7
175.785 mb
175.795 mb
175.79 mb
175.8 mb
ARL10
ARL10
MIR1271
ARL10
ARL10
KIAA1191
KIAA1191
